# Study on the structure-performance relationship between binder types and aluminum-based lithium adsorbent

**DOI:** 10.3389/fchem.2025.1628941

**Published:** 2025-09-26

**Authors:** Ben Ma, Xiaoyu Wang, Jing Zhou, Lijuan Zhang, Ruibin Liu, Li Su, Wenlong Wang, Qinglei Wang, Ping Li, Xuehui Shangguan, Faqiang Li

**Affiliations:** 1 School of Chemistry & Chemical Engineering, Linyi University, Linyi, China; 2 School of Materials Science and Engineering, Linyi University, Linyi, China; 3 School of Chemistry and Materials Science, Qinghai Minzu University, Qinghai, China; 4 Shandong Xinhai Technology Co., Ltd., Linyi, China

**Keywords:** aluminum-based lithium adsorbents, granulation, binders, adsorption-desorption, salt lake

## Abstract

Aluminum lithium layered double hydroxides adsorbents (Li/Al-LDH) are used in industry due to their mild adsorption/desorption conditions, good stability and low cost. However, traditional powdered aluminum lithium adsorbents exhibit poor fluidity and a relatively high dissolution rate. The granulation strategy using binders is employed to address the aforementioned challenges. Nevertheless, there is a lack of systematic research on the relationship between the type of binder and the adsorption and desorption efficiency, as well as the kinetics and thermodynamic mechanisms of mass transfer. This work focuses on the structure-activity relationship between adsorbents and three binders (polyvinyl chloride (PVC), polyvinylidene fluoride (PVDF) and calcium alginate (SA)). The experimental results demonstrate that the adsorption/desorption performance of the adsorbent varied significantly with temperature depending on binder type. It is noted that the structures of PVDF-LDH and PVC-LDH changed during temperature changes, resulting in decreased adsorption and desorption performance. While the SA-LDH can maintain good structural stability and adsorption and desorption capabilities. Besides, in 300 ppm LiCl solution, SA-LDH presents a high adsorption and desorption capacity, with the maximum desorption capacity at 40 °C being 5.84 mg/g and the maximum adsorption capacity at 60 °C being 5.67 mg/g. This study elucidates the regulatory mechanisms of temperature on adsorption/desorption behaviors in binder-formulated granulated adsorbents, providing critical insights for optimizing industrial aluminum-based lithium adsorbents in salt lake lithium extraction.

## 1 Introduction

In recent years, the rapid advancement of electronic devices and electric vehicles, coupled with government subsidies for new electronic purchases, has led to a steady increase in global demand for lithium salts (Swain, 2017). Lithium, the lightest metal, is prized for its low electrochemical potential and is a crucial material in battery production (Zhou et al., 2025). Lithium salts also exhibit exceptional stability, making them indispensable in battery technology (Ren et al., 2024; Yang et al., 2024). Currently, the primary sources of lithium salts are salt lake brine and spodumene deposits (Agency, 2024). The “Lithium Triangle”, spanning Bolivia, Chile, and Argentina, contains 55% of the world’s lithium reserves (Zhu et al., 2023). China possesses abundant lithium resources, among which salt lake brines account for more than 60%. However, the poor quality of the resources and the high cost of extraction present significant challenges.

Several extraction techniques, including adsorption (Chen et al., 2020; Zhang X. et al., 2024), membrane filtration (Guo et al., 2016; Peng et al., 2024), and electrochemical methods (Battistel et al., 2020; Liu et al., 2020), have been developed. The adsorption process typically uses old brine and adsorbents to selectively capture lithium ions, separating them from impurities, and elutes the lithium via deionized water or acid. A key challenge is the separation of lithium from magnesium. The adsorption process requires suitable adsorbents, primarily including lithium aluminium-based adsorbents (Li/Al-LDHs), lithium manganese-based adsorbents (LMOs) (Liu et al., 2025), and lithium titanium-based adsorbents (LTOs) (Wei et al., 2020).

Among them, the manganese-based lithium adsorbents are mainly in the form of LiMn_2_O_4_(Chen et al., 2018), Li_1.33_Mn_1.67_O_4_ (Li X. et al., 2024; Zhao M. et al., 2023) and Li_1.6_Mn_1.6_O_4_ (Bao et al., 2022; Ding et al., 2022), while the titanium-based lithium adsorbents are mainly in the structure of Li_4_Ti_5_O_12_(Shoghi et al., 2021) and Li_2_TiO_3_(Yang Z. et al., 2022). The primary challenge of manganese-based lithium adsorbents is manganese depletion. This stems from the Jahn-Teller effect in Mn^3+^, which damages the lattice and lowers capacity (Liu et al., 2021). Research shows that heteroatom doping stabilizes the spinel structure because these elements form stronger bonds with Mn or O (Gao et al., 2023). Compared to LMOs, LTOs exhibit a similar adsorption mode and high capacity while enhancing structural stability through Ti-O bonds that reduce metal loss. A trade-off exists between structural stability and ion mobility. Although LTOs offer greater durability, longer service life, and enhanced safety, they sacrifice some lithium mobility relative to LMOs, resulting in slower extraction kinetics (Zhao B. et al., 2023). Optimizing the material’s structure can address this issue. Among them, only lithium aluminium-based adsorbents are widely used in commercial applications, though their adsorption capacity is relatively low (Zhang et al., 2023).

Manganese-based and titanium-based lithium adsorbents exhibit poor stability during industrial use, with significant material loss and low reusability, reducing their effectiveness in repeated applications. The desorption process relies on acid, which pollutes the brine and harms the environment. Waste acid disposal further worsens the ecological impact. Aluminum-based lithium adsorbents are widely used in the industry due to their low dissolution loss and environmental benefits (Yu et al., 2022). During industrial adsorption-desorption processes, preventing excessive Li^+^ desorption is challenging, especially when adjustments to desorption conditions are required due to changes in process parameters or brine compositions. These changes often lead to the collapse of the LDH structure, reducing its Li^+^ adsorption capacity.

To tackle this problem, many studies have explored doping as a strategy to enhance LDH stability (Luo et al., 2022). However, powdered adsorbents can cause blockages in industrial adsorption towers due to high pressure and brine volume, while also being prone to loss during extraction. Granulation methods, such as wet, dry, and extrusion granulation, address these issues by mixing adsorbents with organic or inorganic binders (Zhang R. et al., 2024). Common binders include PVC (Pan et al., 2024), PVDF (Li H. et al., 2024), PAN (Wang et al., 2020), PVA (Zhao M. et al., 2023), and calcium alginate formed by cross-linking sodium alginate with calcium chloride (Tian et al., 2023). Nevertheless, there is a lack of systematic research on the relationship between the type of binder and the adsorption and desorption efficiency, as well as the kinetics and thermodynamic mechanisms of mass transfer.

In this work, urea hydrolysis is employed to directly synthesize aluminium-based lithium adsorbent (LDH). The wet granulation method is then explored to form LDH granules using three cross-linking agents: PVC, PVDF, and SA. The aim is to enhance the industrial applicability of the adsorbent by doping the powder with these agents. The SEM, XRD, FT-IR, and TEMare carried out to research the structure-activity relationship between adsorbents and three binders (PVC, PVDF and SA). This research offers valuable insights for the commercial use of aluminium-based lithium adsorbents in lithium extraction from salt lakes.

## 2 Experimental

### 2.1 Materials and methods

AlCl_3_·6H_2_O (AR, 97.0%), LiCl (anhydrous, AR, ≥99.0% (AT)), Urea (AR, 99%), CaCl_2_ (AR, 96.0%), and Sodium alginate (AR) are purchased from Shanghai Aladdin Biochemical Technology Co. Ltd. All chemicals are used without further purification.

### 2.2 Synthesis of aluminium-based lithium adsorbents

A mixture of AlCl_3_·6H_2_O, LiCl and Urea is dissolved in water and subsequently transferred to a three-neck flask. The reaction is conducted in the flask, heated to 90 °C, and stirred at 300 rpm for 24 h. Upon completion of the reaction, the mixture is aged for 16 h. Following the aging process, the mixture in the flask develops into stratified layers, with a clarified upper layer and a dense white precipitate in the lower layer. The components are subsequently separated in a centrifuge and thoroughly washed with deionized water. Following rinsing, the material is oven-dried and subsequently ground into a fine powder. Among them, the molar ratio of the Al(OH)_3_, urea, and LiCl is 1:10:3.

### 2.3 Granulation process

The dried Layered Double Hydroxide (LDH) powder is thoroughly mixed with an 2wt% aqueous sodium alginate solution under vigorous stirring to form a uniform suspension. This suspension is then slowly dripped into an 4wt% aqueous calcium chloride solution, initiating cross-linking between the sodium alginate and calcium ions, which resulted in the formation of numerous gel-like pellets at the bottom of the solution. The pellets are left to mature in the calcium chloride solution for 24 h, and washed with deionized water to remove any residual calcium ions. In a similar manner, a specified quantity of LDH powder is sufficiently blended with 6.7wt% polyvinyl chloride (PVC) dissolved in N-methyl-2-pyrrolidone (NMP) solution. Then, the aforementioned precursor solutionis slowly dripped into deionized water, whereupon it gives rise to numerous spherical particles at the bottom of the solution. This is because that NMP and water are miscible in any proportion, facilitating the formation of the spheres. Subsequently, the particles are collected by filtration and thoroughly rinsed with deionized water to wash off any residual solvent. The granulation process for polyvinylidene fluoride (PVDF) is analogous to that of PVC. It is noted that all the mass fraction ratio of adsorbent to binder is 4:1. and the above adsorbent is named SA-LDH, PVC-LDH and PVDF-LDH, respectively. An adsorbent-to-binder mass ratio of 4:1 was employed to ensure a rigorous comparative evaluation of the adsorption and desorption performance of the three granulation techniques and to maximize active adsorbent loading in each granule (Supplementary Figures S1, S2).

### 2.4 Adsorption experiments

Static adsorption experiments are conducted on LDH, LDH-PVC, LDH-PVDF, and LDH-SA samples in a temperature-controlled air oscillator running at 200 rpm with a solid-liquid ratio of 1 g/100 mL. The adsorption–desorption cycle lasted 4 h, during which lithium equilibrium is achieved under controlled temperatures and constant agitation. Samples are collected at regular intervals for elemental analysis, and the tests are performed at preset temperatures of 20 °C, 40 °C, and 60 °C.

Li^+^ desorption capacity (Qe) is calculated according to formula 1:

$$\text{Qe}=\frac{\text{Ce}*V}{m}$$

where Ce (mg/L) is the concentration of lithium ions in the filtrate after filtration; V(L) is the volume of the filtrate; m(g) is the mass of the adsorbent.

Li^+^ adsorption capacity (Qe) is calculated according to formula 2:

$$\text{Qe}=\frac{C_{0}‐C_{t}}{m}V$$

Where C_0_ (mg/L) is the concentration of lithium ions in the brine at the initial time; C_t_ (mg/L) is the concentration of lithium ions in the solution after adsorption at time t. V(L) is the volume of the filtrate; m(g) is the mass of the adsorbent.

The kinetics of the process is described using pseudo-first-order kinetic models and pseudo-second-order kinetic models.

$$\frac{{\text{dq}}_{t}}{\text{dt}}{=k}_{1}\left(q_{e1}{‐q}_{t}\right)$$

tq t=1k2·q2 e2+1q e2·t

where q_e1_ (mg/g) and q_e2_ (mg/g) are the amount of lithium adsorbed at equilibrium. q_t_ (mg/g) is the amount of lithium adsorbed at moment t. k_1_ [g/(mg⋅min)] and k_2_ [g/(mg⋅min)] are adsorption rate constants.

## 3 Results and discussion

### 3.1 Preparation and structural characterization

**FIGURE 1:**
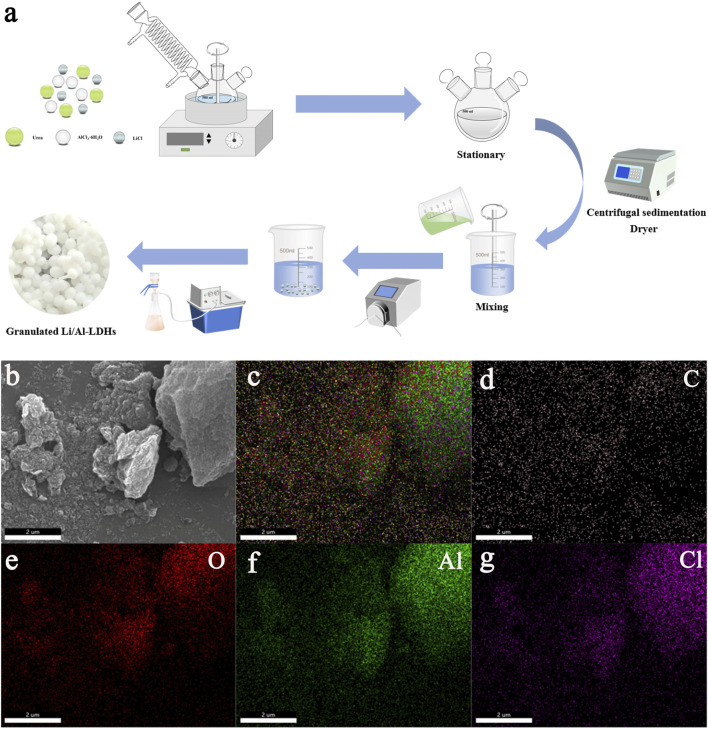
**(a)** Synthesis schematic diagram of LDH. **(b)** SEM image of LDH. **(c–g)** The EDS elemental mapping images of LDH.

Figure 1a shows a simplified flow chart of aluminum-based lithium adsorbent LDH and granulation. Aluminum hydroxide, urea and lithium chloride are used to synthesize aluminum-based lithium adsorbent LDH by a one-pot method, and different adhesives are added for wet granulation. The SEM images reveal that the LDH powder exhibits a layered porous morphology (Figure 1b). Furthermore, EDS mapping confirms the uniform distribution of Al, Cl, O, and C elements throughout the sample (Figures 1c–g), indicating that the reactants have undergone complete reaction.

**FIGURE 2:**
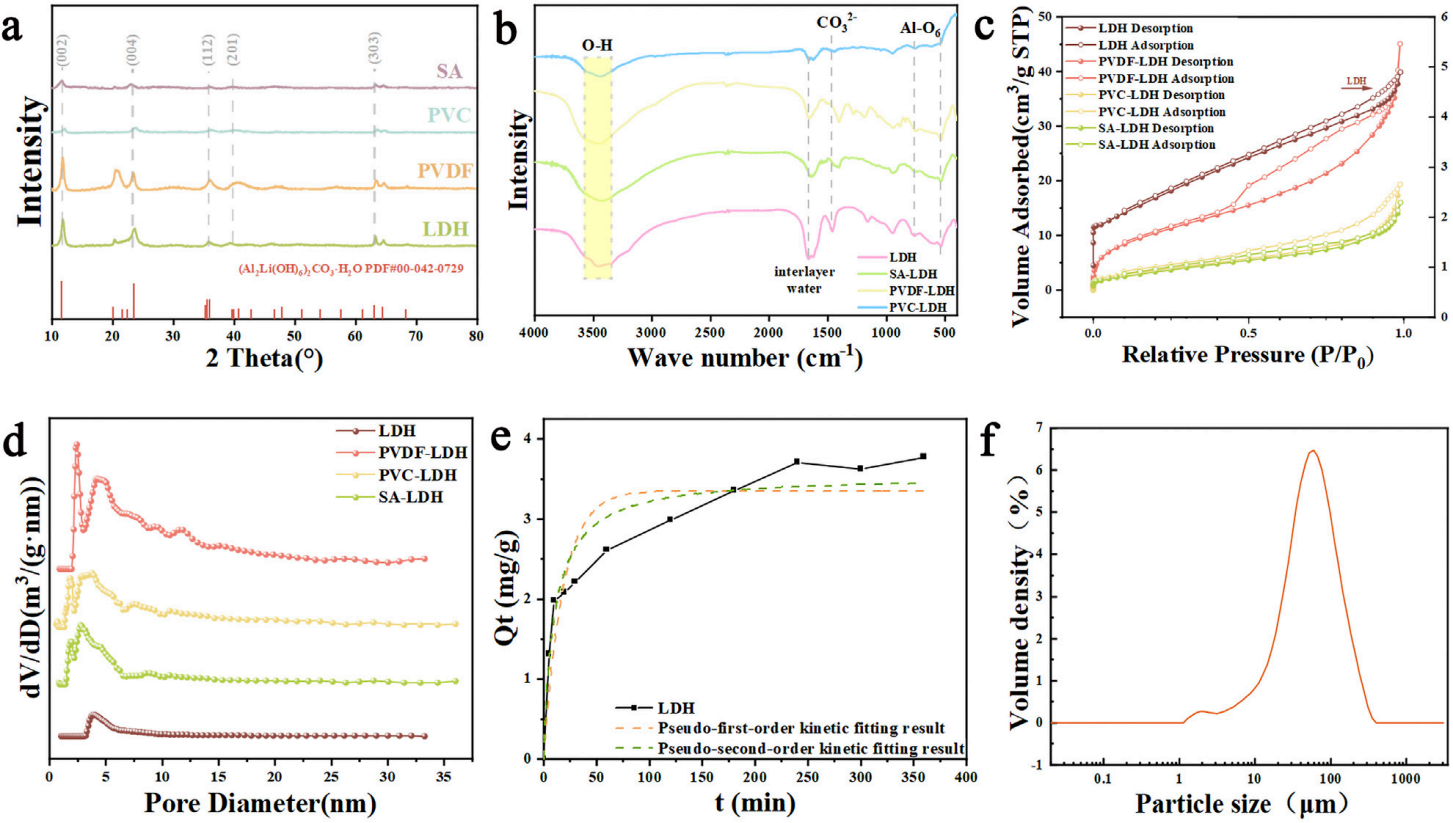
**(a)** XRD **(b)** FTIR **(c)** N_2_ adsorption–desorption isotherms **(d)** pore size distribution of LDH, PVC-LDH, PVDF-LDH and SA-LDH. **(e)** Adsorption isotherm fitting results **(f)** Particle size distribution of LDH.

Figure 2a presents the XRD patterns of LDH powder and its granulated forms using three distinct methods. The primary crystalline phase of LDH powder is identified as (Al_2_Li(OH)_6_)_2_CO_3_·H_2_O with the standard crystal positions such as (002), (004), (006), (112), (201) and (303). And all three granulation methods distinctly preserve the lithium-extracting component of LDH. It is worth noting that PVDF, a semicrystalline polymer with a crystallinity of approximately 50%–65%, exhibits a pronounced and sharp peak at 20 ° (Supplementary Figure S4), which enhances the characteristic diffraction peaks of the LDH structure. In contrast, PVC, as a polymer with limited crystallinity, generates an amorphous scattering signal that significantly intensifies in the range of 15 °–20 ° (Supplementary Figure S5), potentially masking weaker diffraction peaks of LDH. Similarly, the amorphous scattering contribution of calcium alginate can obscure certain cut down LDH diffraction peaks. TEM reveals a petal like agglomerate morphology composed of tightly packed layers, and clearly shows its two-dimensional structure, hexagonal nanosheet structure. In the HRTEM image (Supplementary Figure S6), continuous lattice fringes with a spacing of 0.76 nm are observed, matching the (002) reflection in the standard PDF card (PDF#00-042-0729). And these data confirm the successful synthesis of Li/Al-LDH. As shown in Figure 2b, the infrared spectrum reveals a broad and intense absorption peak at 3,460 cm^-1^ (Benhiti et al., 2020), corresponding to the superimposed hydroxyl stretching vibrations (-OH) from hydroxyl groups within the LDH hydrotalcite layers and hydrogen-bonded interlayer water molecules. A distinct band at 1,642 cm^-1^ is attributed to the vibrations of interlayer water molecules, while the absorption at 1,463 cm^-1^ is belong to carbonate group vibrations (Stoica and Pérez-Ramírez, 2007). Additionally, the Al–O vibrations, influenced by interlayer anions, displays a peak around 530 cm^-1^ (Chen et al., 2021), with slight shifts in position due to these interactions. Notably, the polar nature of PVC and PVDF polymers results in strong infrared absorption, leading to relatively low transmittance in the IR spectrum (Supplementary Figures S7, S8).

The XPS survey spectrum (Supplementary Figure S10) displays strong Al 2p and O 1s signals; the Li 1s signal is weak due to its low surface concentration, and no nitrogen peak is detected—indicating effective washing. Cl 2p signal remains, which is attributed to residual LiCl and AlCl_3_·6H_2_O from the precursor. The Raman spectrum (Supplementary Figure S11) exhibits three characteristic LDH bands at 316 cm^-1^, 396 cm^-1^, and 565 cm^-1^, further validating the layered double hydroxide structure. A typical crystal structure of LDH is illustrated in Supplementary Figure S12.

Nitrogen adsorption-desorption isotherms are carried out to analyzed the pore structural characteristics of LDH, PVDF-LDH, PVC-LDH, and SA-LDH (Figure 2c). All samples display type IV isotherms, with the absence of a clear saturated adsorption plateau, reflecting their highly irregular pore structures. The H3-type hysteresis loop observed in the isotherms is indicative of a layered mesoporous framework, which is further corroborated by pore size distribution analysis. Notably, the adsorbents subjected to wet granulation exhibit an increased BET specific surface area and a larger pore volume (Figure 1d). This suggests that the granulation process facilitates the formation of additional micropores and mesopores, which are favorable for enhanced lithium-ion transport.

Figure 2e and Supplementary Table S1 present the adsorption rate obtained by fitting the kinetic equation of the aluminum-based lithium adsorbent powder. The pseudo-first-order kinetic model is fitted to obtain k_1_ of 0.3990 and the pseudo-second-order kinetic model is fitted to obtain k_2_ of 0.0158 (Figure 2e). Both sets of parameters indicate that during the adsorption process, the exchange rate of substances in the adsorbent powder is small, and the adsorption reaction as a whole presents a relatively slow characteristic. According to the linear fitting degree R_2_ of the two models in Supplementary Table S1, it can be concluded that the pseudo-second-order kinetic model is better than the pseudo-first-order kinetic model in fitting effect.

LDH reveals a broad pore size distribution, which demonstrates the formation of phase-separated structures during the synthesis process. The lamellar framework serves as a pathway for lithium ion adsorption and desorption, while the porous structure functions as a reservoir for lithium storage. The particle size distribution analysis manifests that the LDH powder possesses a uniform and homogeneous particle size (Figure 2f). Additionally, numerous hydrogen bonds are present both within the LDH structure and between LDH and water molecules. Among them, the average particle size of LDH powder was 57.9 μm (Supplementary Table S2).

**FIGURE 3:**
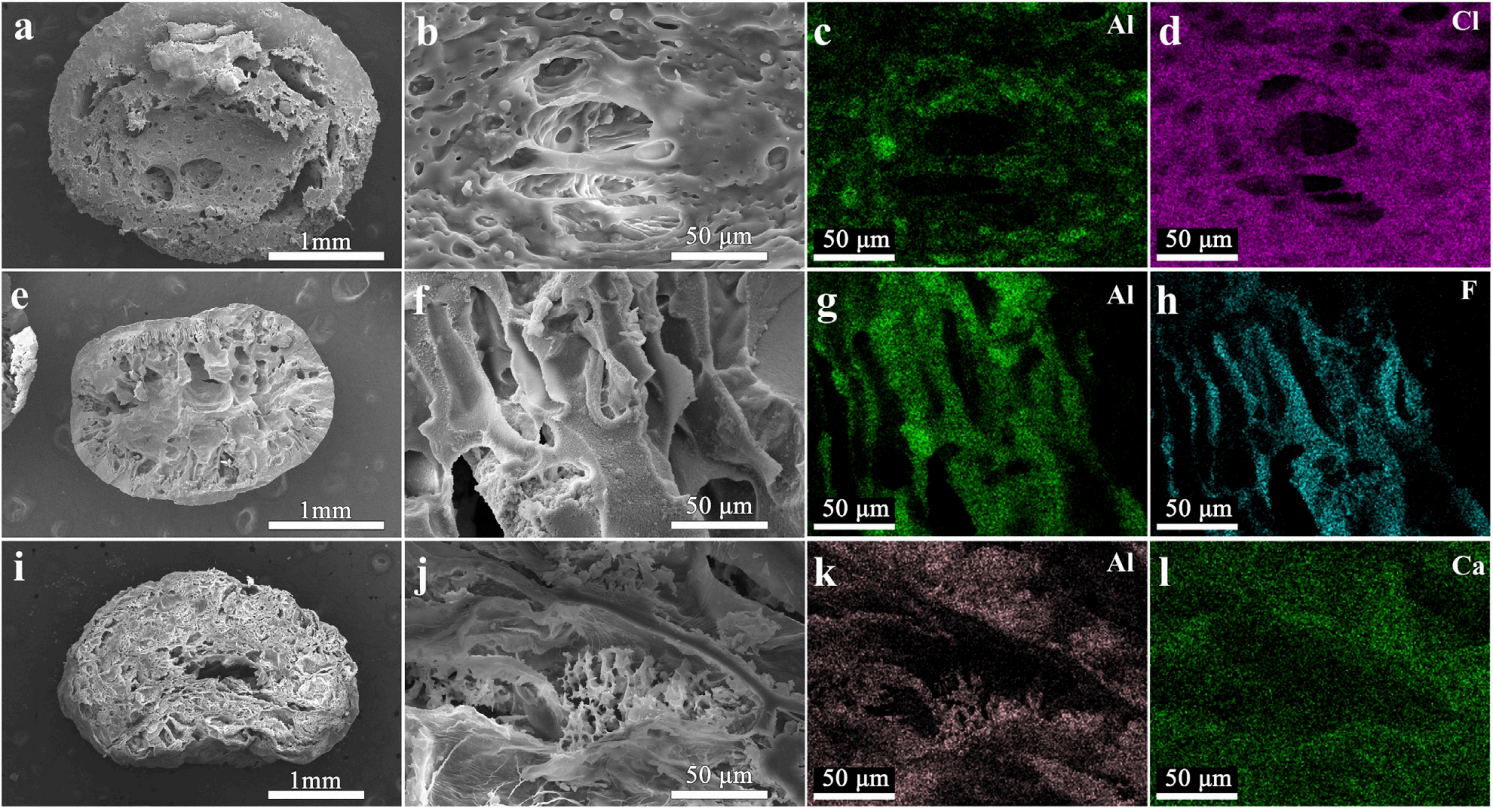
SEM images of PVC-LDH, PVDF-LDH and SA-LDH. Macroscopic cross section of **(a)** PVC-LDH **(e)** PVDF-LDH **(i)** SA-LDH Microstructure of **(b)** PVC-LDH **(f)** PVDF-LDH **(j)** SA-LDH. Characteristic elements of EDS spectrum in **(c,d)** PVC-LDH **(g,h)** PVDF-LDH **(k,l)** SA-LDH.

Figure 3 illustrates the SEM patterns of LDH, PVDF-LDH, PVC-LDH, and SA-LDH before and after cycling, alongside the EDS elemental mapping distribution images captured prior to cycling. Increasing the binder proportion may embed some adsorption sites in LDH, rendering them inactive for adsorption, while the dense membrane structure obstructs ion diffusion and interaction with LDH powder (Gregorio and Cestari, 1994). This hinders lithium ion adsorption, ultimately decreasing the adsorption capacity of Li^+^ during granulation. Hence, a 1:4 ratio of binder to adsorbent is employed in this experiment.

Granulation via titration result in particles approximately 1–2 mm in size, featuring smooth surfaces covered by a uniform film, significantly minimizing powder leakage. These granules are easy to filter and maintain shape during agitation and rinsing. Differences between PVC, PVDF-LDH, and SA-LDH samples are evident. Post-granulation, the binders formed a mesh structure inside the granules, integrating the adsorbent powders and providing structural stability. Pore size and quantity vary with binder type, facilitating adsorption site exposure and lithium ion transfer, although excessive porosity may destabilize the granules. It can be seen from the EDS element mapping distribution image that the LDH powder has been completely and evenly distributed in PVDF, PVC and SA.

### 3.2 Absorption and desorption properties

**FIGURE 4:**
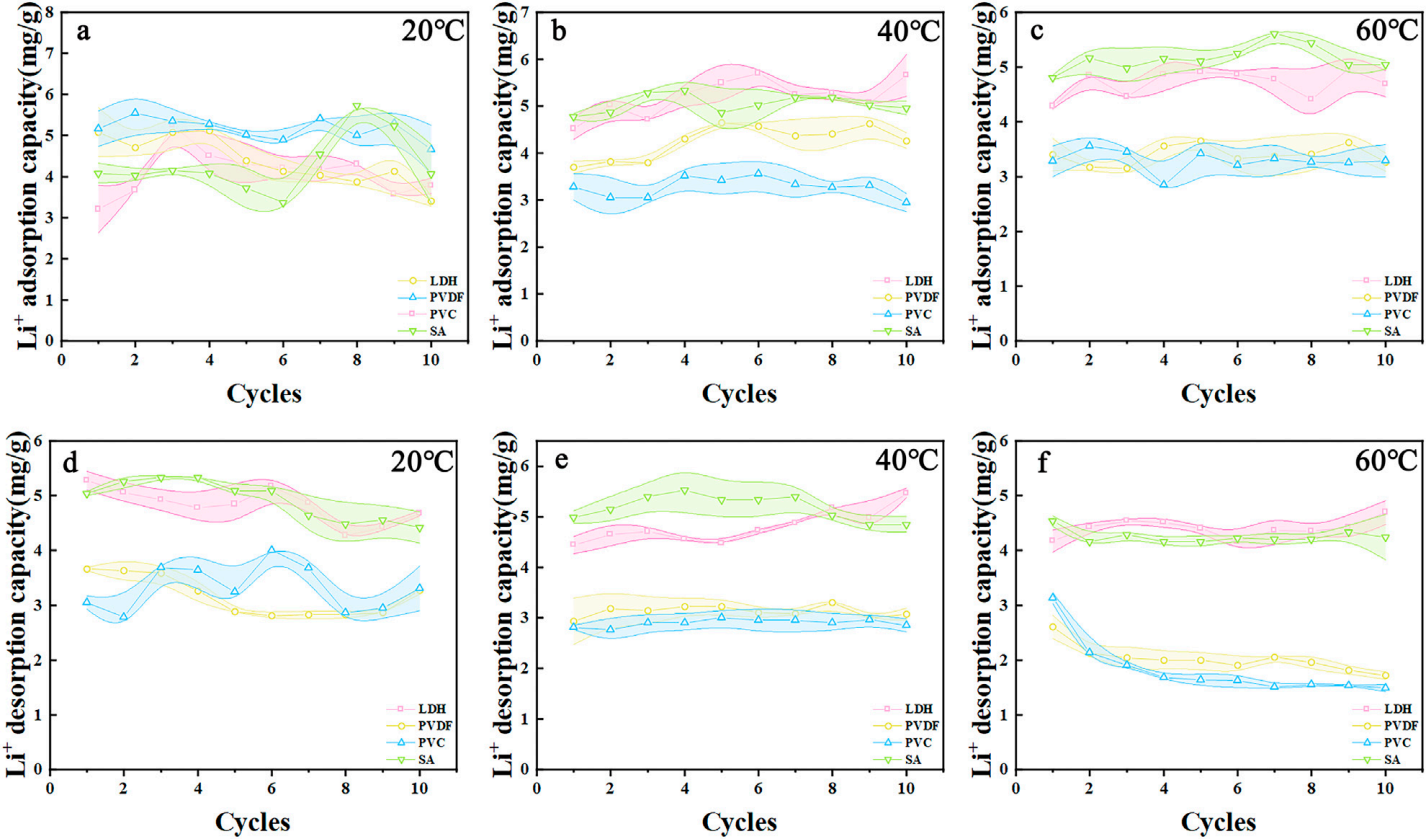
Adsorption capacity of LDH, PVC-LDH, PVDF-LDH and SA-LDH at **(a)** 20 °C **(b)** 40 °C **(c)** 60 °C. Desorption capacity of LDH, PVC-LDH, PVDF-LDH and SA-LDH at **(d)** 20 °C **(e)** 40 °C **(f)** 60 °C.

The adsorption performance of different LDH shows minimal variation across different temperatures (Figure 4). In contrast, the adsorption capacity of PVC and PVDF granules decreases with increasing temperature, with repeated adsorption cycles even leading to negative adsorption capacities. During the desorption process, LDH demonstrates the most effective lithium-ion removal at 40 °C, achieving a desorption capacity of 5.47 mg/g (Figure 4e), whereas its performance is weakest at 20 °C (Figure 4d). SA-LDH exhibits relatively consistent lithium removal performance across temperatures, with a maximum desorption capacity of 5.84 mg/g, though its capacity is slightly reduced at 60 °C with a maximum adsorption capacity of 5.67 mg/g at 60 °C (Figure 4c). PVC-LDH and PVDFLDH show similar desorption efficiencies at 20 °C and 40 °C; however, their performance declines sharply at 60°C. At the same temperature, LDH raw powder and SA-LDH exhibit superior lithium adsorption and desorption capacities compared to PVC-LDH and PVDF-LDH, particularly at 40 °C and 60 °C.

However, practical considerations for industrial applications must account for the particle size of LDH raw powder, which is relatively small. During brine flow, the fine LDH particles tend to accumulate densely, reducing porosity, leading to local agglomeration, and potentially causing blockages. In industrial settings, granulated adsorbents are uniformly packed and flow through adsorption columns. The long-term mechanical strength of granulated adsorbents is critical for sustained industrial use (Li et al., 2023). While SA offers high adsorption and desorption capacities, its mechanical strength is lower than that of PVC and PVDF (Supplementary Figure S13). Consequently, PVC and PVDF granules, with their superior mechanical properties, may be more suitable for widespread industrial applications.

### 3.3 Characterization of LDHs after cycling

**FIGURE 5:**
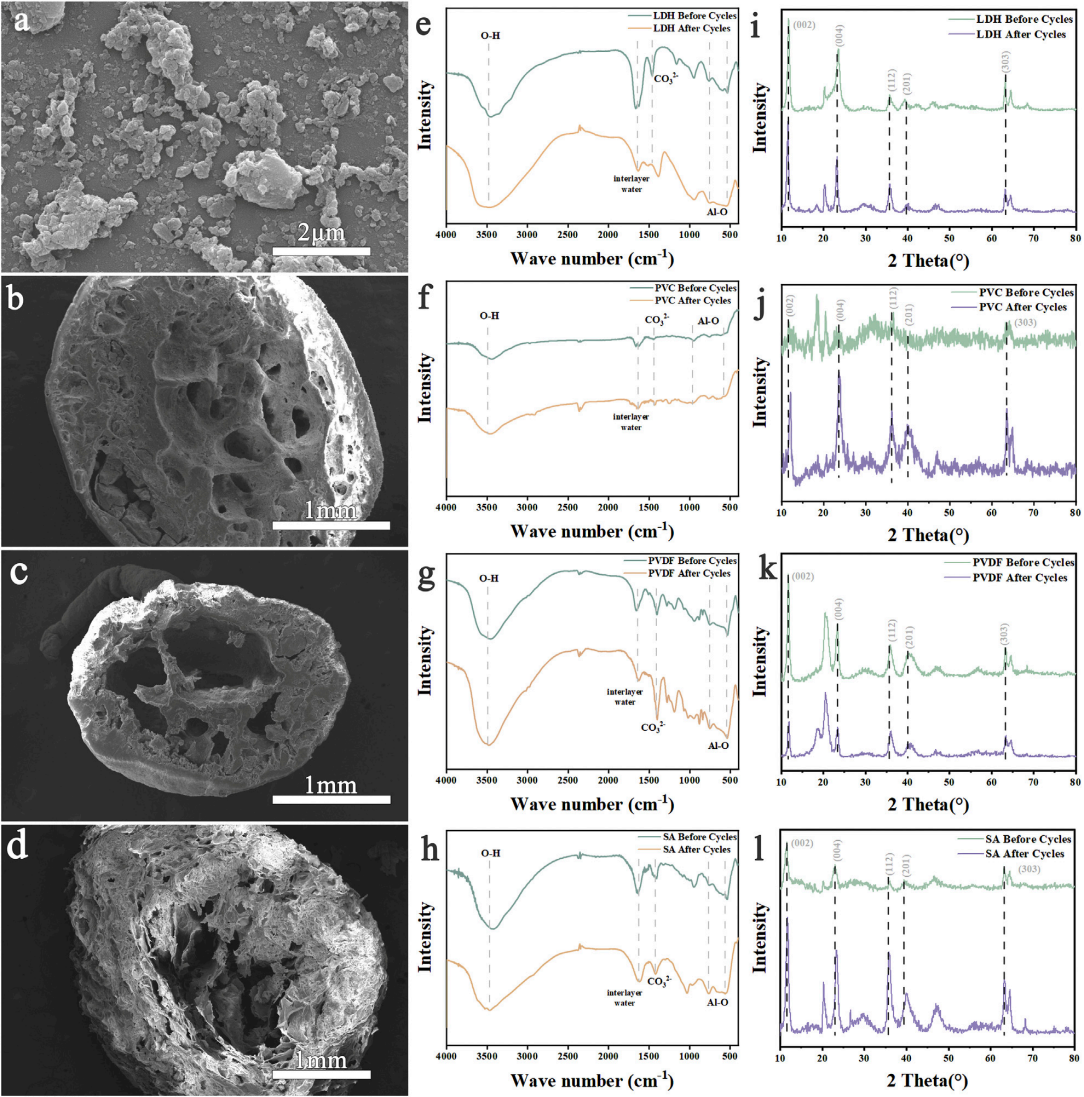
SEM images of **(a)** LDH, **(b)** PVC-LDH, **(c)** PVDF-LDH and **(d)** SA-LDH after cycling. FTIR comparison of **(e)** LDH, **(f)** PVC-LDH, **(g)** PVDF-LDH and **(h)** SA-LDH before and after cycling. XRD comparison of **(i) **LDH, **(j)** PVC-LDH,** (k)** PVDF-LDH and **(l)** SA-LDH before and after cycling.

Figure 5a presents the SEM images of LDH After ten cycles, the structure of the LDH raw powder changed noticeably. The CO_3_^2-^ peak shifted from 1,463 cm^-1^ to 1,387 cm^-1^, and the Al-O peak became flatter, though it remained detectable. After ten adsorption-desorption cycles of granulated LDH, some surface structural changesare observed, but the adsorption performance remained largely unaffected. The XRD spectrum reveals that gibbsite peaks emerge after ten cycles of LDH, and the characteristic peaks of LDH become sharper. However, the gibbsite peak remains relatively indistinct. The high ductility of PVC-LDH makes it difficult to grind into powder, resulting in slightly noisy XRD signals. Despite this, both LDH and gibbsite peaks are still clearly visible. After the PVDF-LDH cycle, gibbsite peaks also appear, partially overlapping with a peak near 20.3 °, exhibiting notable sharpness. In contrast, after ten cycles of SA-LDH granulated with calcium alginate, the gibbsite peak intensity is weaker than that of PVC-LDH and PVDF-LDH, with less distinct peaks.

The hydrophobic nature of PVC and PVDF likely minimizes their impact on the hydration environment of the adsorbent surface (Meringolo et al., 2018; Zhang et al., 2020). During the adsorption-desorption cycle, aluminum-based adsorbents are more prone to hydration in aqueous environments, increasing the formation of gibbsite. Calcium alginate, being highly hydrophilic, forms a stable network with water, potentially reducing the contact between aluminum-based adsorbents and water. This property slows the hydration reaction and limits gibbsite formation. Additionally, the polycarboxyl groups in calcium alginate may chemically bond with aluminum-based adsorbents, forming stable complexes that prevent lattice reconstruction and crystal collapse. Together, these factors reduce gibbsite generation. The infrared spectra showed no significant structural changes in the three types of granulated LDH, confirming that their intrinsic structure remained stable. XRD analysis revealed peaks of alumina trihydrate in all samples. The adsorption properties of PVC-LDH, PVDF-LDH, and SA-LDH at 20 °C showed minimal variation. After ten cycles, the aluminium ion content in the desorption solution was measured. The LDH raw powder exhibites partial structural collapse and dissolution loss, whereas the aluminium loss in pelletized PVC-LDH, PVDF-LDH, and SA-LDH is significantly lower. This indicates that granulation improved the stability of the aluminium-based lithium adsorbent particle structure, reducing particle abrasion. Granulation also altered the adsorbent’s surface area and pore structure, making the particles more regular and well-proportioned. These changes contributed to more stable adsorption performance and further reduced aluminium loss.

**FIGURE 6:**
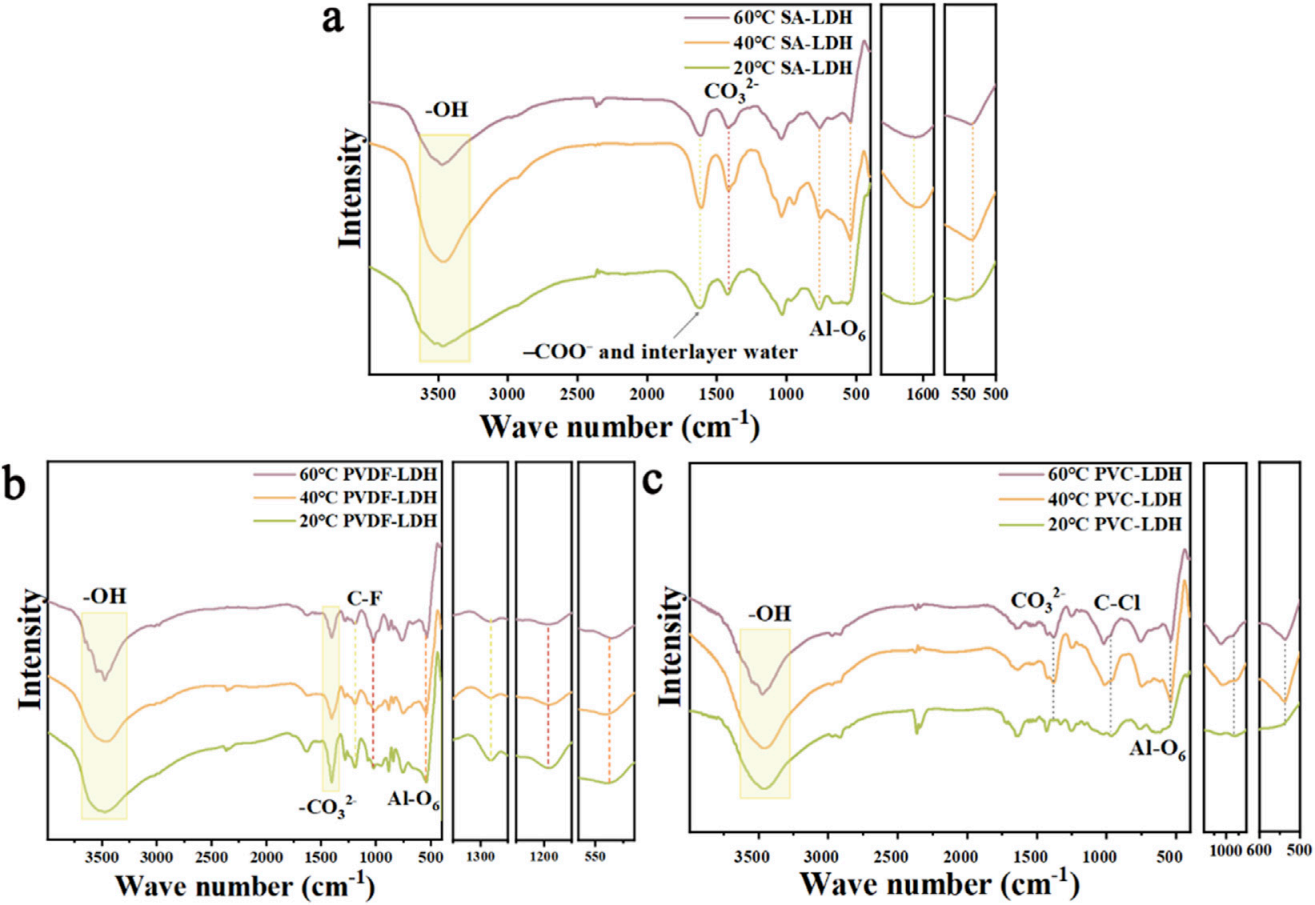
Comparison of infrared spectra of **(a)** SA-LDH, **(b)** PVDF-LDH and **(c)** PVC-LDH after cycling at different temperatures.

Figure 6 presents the FTIR spectra of SA-LDH, PVC-LDH, and PVDF-LDH after ten adsorption–desorption cycles at 20 °C, 40 °C, and 60 °C. In Figure 6a, the main differences observed for SA-LDH at different temperatures include a progressive narrowing of the broad hydroxyl absorption band (3,600–3,400 cm^-1^) and the carbonate band associated with free CO_3_^2-^ ions at 1,385 cm^-1^ as the temperature increases. Additionally, the peak originally observed at 969 cm^-1^ disappears at 60 °C, likely due to the combined effects of calcium alginate and LDH interactions (Tian et al., 2023). Although slight changes occur in the Al-O_6_ octahedral vibration and stretching band at 540 cm^-1^, these alterations do not impact the material’s adsorption–desorption performance.

Figure 6b also shows that the wide absorption band of hydroxyl groups in the aluminum hydroxide layer at 3,600–3,400 cm^-1^ becomes narrower with increasing temperature. When the temperature rises, some hydrogen bonds may break or reconfigure, causing the local environment where the hydroxyl groups are located to become more uniform, the structural order is improved, and the molecular vibration coupling effect is weakened, which ultimately leads to a narrowing of the absorption peak. At 1,385 cm^-1^ is the CO_3_^2-^ in the carbonate-type aluminum-based lithium adsorbent. A moderately strong CF_2_ vibration peak appears near 1,419 cm^-1^, C-F stretching and bending vibrations near 1,280 cm^-1^, and there are also multiple strong C-F vibration peaks in the 1,165 cm^-1^ region. When the temperature rises, the molecular chain of PVDF will gain more thermal energy, and its segment motion and conformation distribution will become more extensive, resulting in a more dispersed distribution of molecular vibration energy levels (Yang J. et al., 2022). This dispersion effect will cause the originally concentrated vibration peak to expand into a wider and blunter absorption band. And when the temperature rises, the local hydrogen bond network, interlayer spacing and hydration state in the aluminum-based lithium adsorbent layer will change, which will cause the chemical environment around the carbonate anion to become chaotic, and then its characteristic vibration peak will expand and become blunt. This leads to a decrease in the adsorption and desorption capacity of PVDF-LDH at elevated temperatures. The peak of Al-O_6_ octahedral vibration and stretching vibration at 547 cm^-1^, the Al-O bond length and bond angle distribution in the octahedron are relatively wide, resulting in a larger distribution of vibration frequencies and a wider absorption peak. When the temperature rises, the octahedral structure becomes more uniform, so that the energy level distribution of Al-O vibration will converge, and the absorption peak will naturally become narrower.

Figure 6c shows the FTIR spectra of PVC-LDH after ten cycles at 20 °C, 40 °C, and 60 °C. The broad hydroxyl absorption band (3,600–3,400 cm^-1^), attributed to the aluminum hydroxide layers in LDH, narrows progressively with increasing temperature, indicating reduced hydrogen bonding or structural rearrangement. At 20 °C, the characteristic peaks of CO_3_^2-^ (1,385 cm^-1^) and Al-O_6_ (547 cm^-1^) are superimposed on the LDH’s original signals. However, at 40 °C and 60 °C, these LDH-specific peaks become distinguishable, suggesting enhanced molecular mobility within the PVC matrix. This increased mobility likely facilitates partial relaxation or reorganization of the polymer chains, diminishing the overlap and allowing the intrinsic LDH peaks to re-emerge (Lin et al., 2005). Additionally, the C-Cl out-of-plane bending vibration at 966 cm^-1^ becomes sharper and shifts with rising temperature. Elevated temperatures promote segmental motion of the PVC chains, reducing conformational diversity and local stress variations (Rao et al., 2023). Consequently, the vibrational frequencies of identical functional groups become more uniform, resulting in narrower and more defined absorption peaks. This factor may lead to a decrease in the adsorption and desorption capacity of PVC-LDH at elevated temperatures. Therefore, rising temperatures significantly reduce the adsorption and desorption capacities of PVDF-LDH and PVC-LDH aluminum-based lithium adsorbents that use organic binders, whereas SA-LDH, which uses calcium alginate as a binder, retains relatively high performance under the same conditions.

## 4 Conclusion

In this study, aluminum-based lithium adsorbents are synthesized by urea hydrolysis, and LDH is incorporated into PVC, PVDF and SA by wet granulation. The adsorption and desorption properties of these three granular materials are evaluated. The results show that PVC and PVDF particles exhibit significantly higher mechanical strength than SA particles. The three materials perform similarly at room temperature. However, with the increase of temperature, the adsorption and desorption properties of PVC-LDH and PVDF-LDH decrease, while SA-LDH still maintain excellent adsorption capacity and desorption capacity at 40 °C and 60 °C. At 60 °C, SA-LDH present an adsorption capacity of 5.67 mg/g and a desorption capacity of 4.34 mg/g, with long-term stable adsorption performance. On this basis, the effect of temperature on the binder granulation structure is studied. This study emphasizes the effect of binders on the adsorption and desorption capacity of aluminum-based lithium adsorbents, providing valuable insights for promoting the extraction of lithium resources from salt lakes.

## Data Availability

The raw data supporting the conclusions of this article will be made available by the authors, without undue reservation.
