# Supplementary material for: Study on the structure-performance relationship between binder types and aluminum-based lithium adsorbent
Source: Front Chem. 2025 Sep 26;13:1628941. doi: 10.3389/fchem.2025.1628941 (PMC12511872; doi:10.3389/fchem.2025.1628941)
Supplement: Supplementary file 1 [file DataSheet1.pdf]

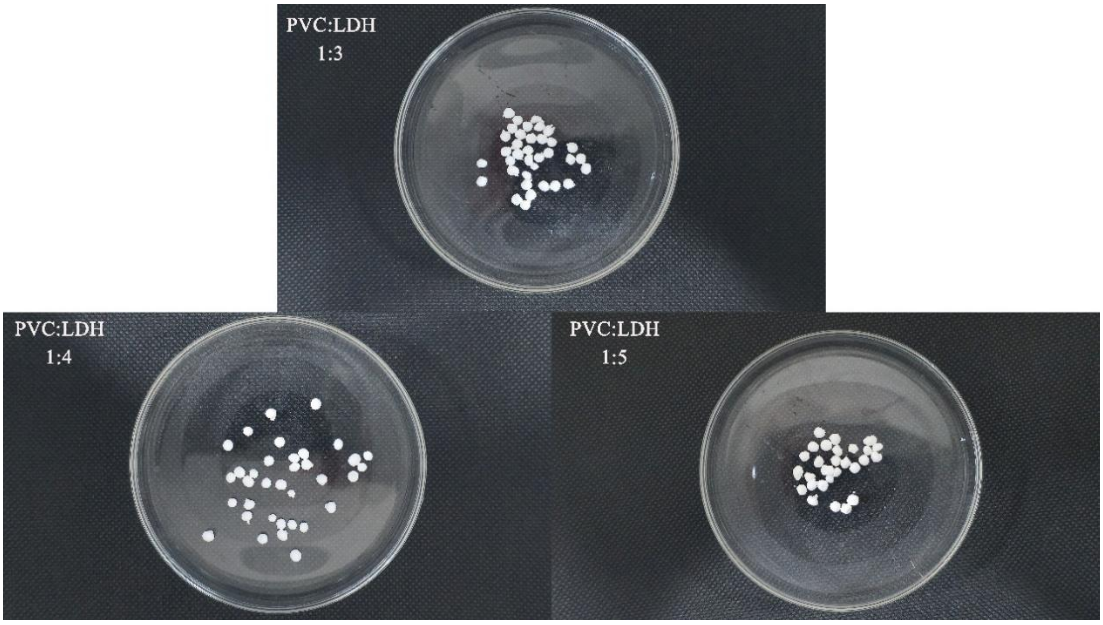

Figure S1. Macroscopic morphology diagram of PVC-LDH.

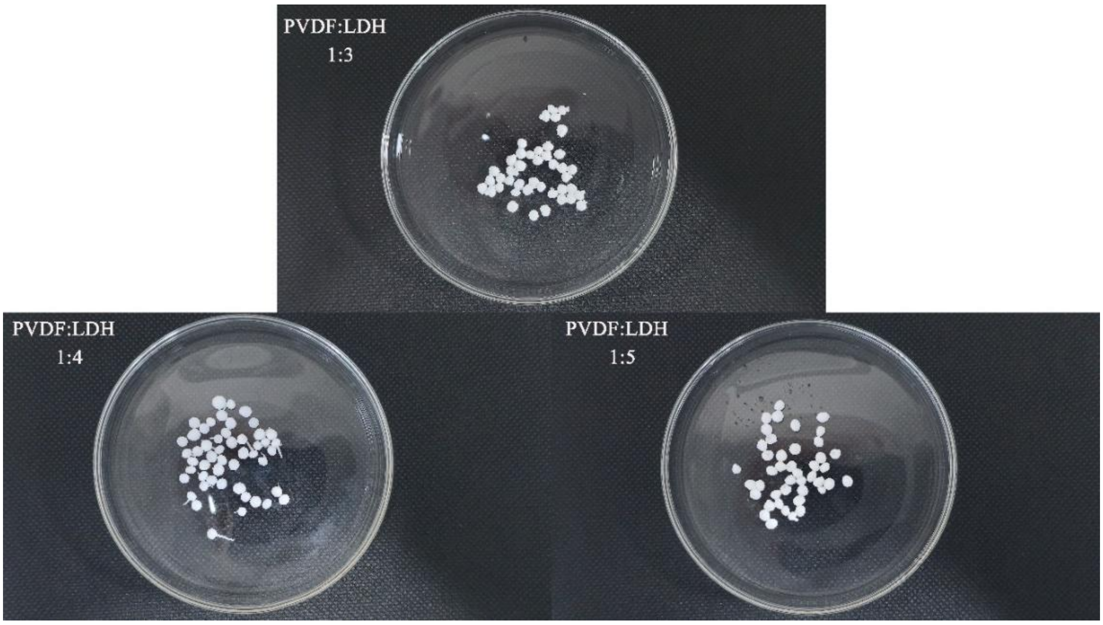

Figure S2. Macroscopic morphology diagram of PVDF-LDH.

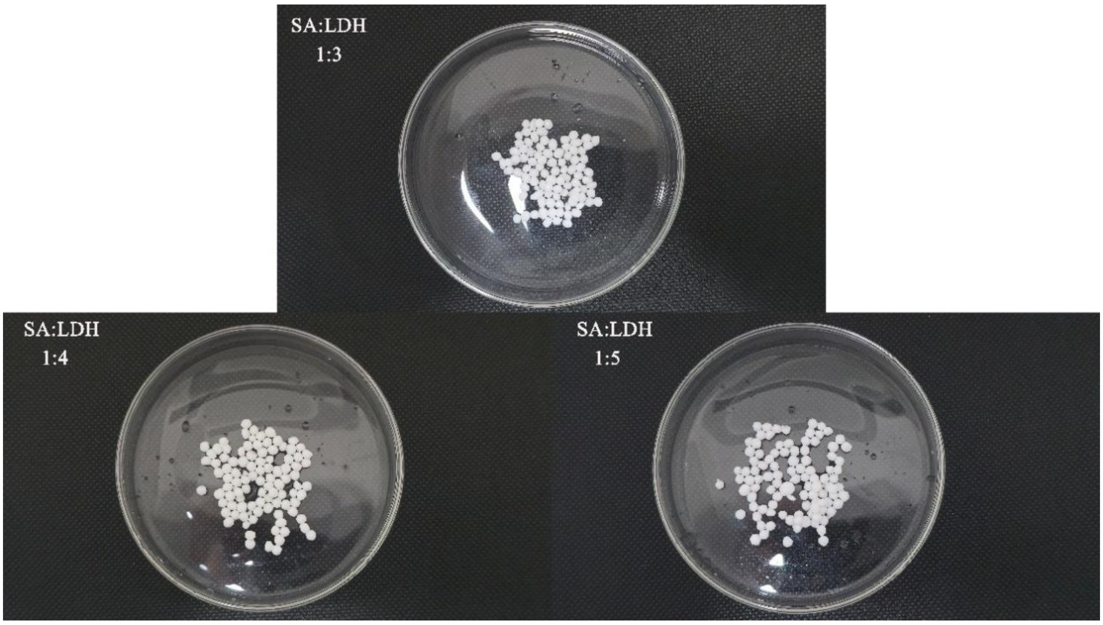

Figure S3. Macroscopic morphology diagram of SA-LDH.

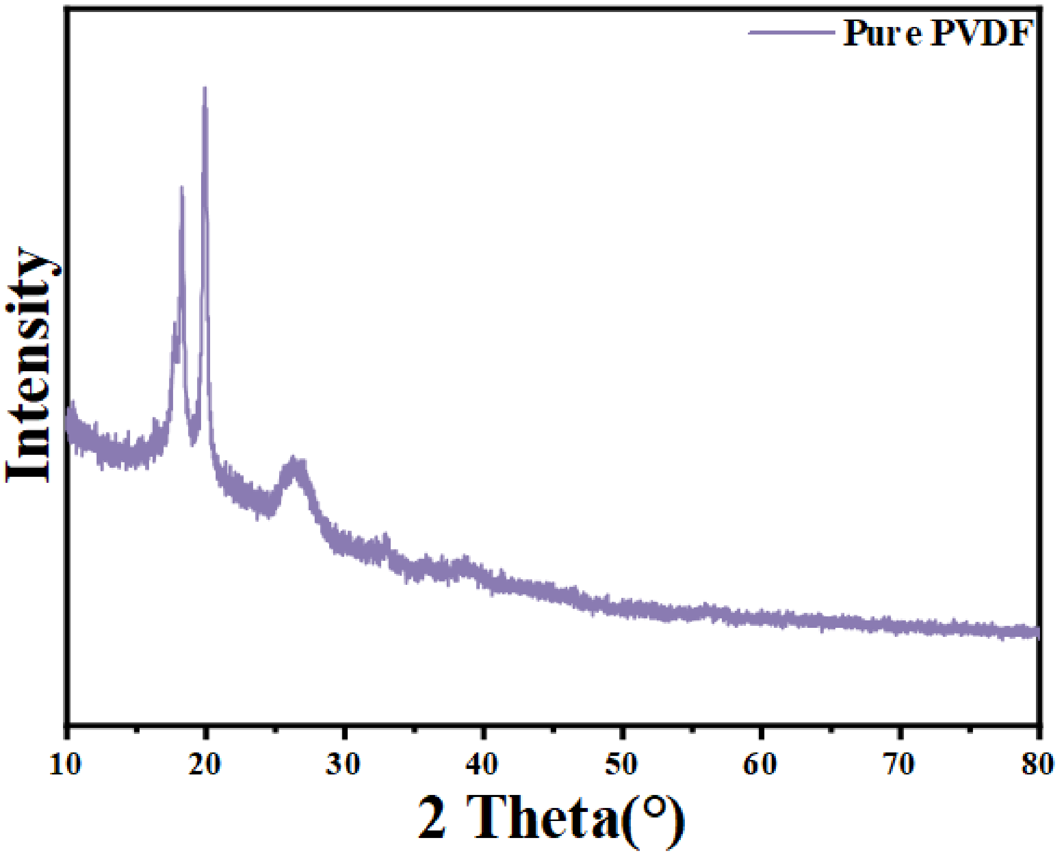

Fig.S4. XRD patterns of PVDF

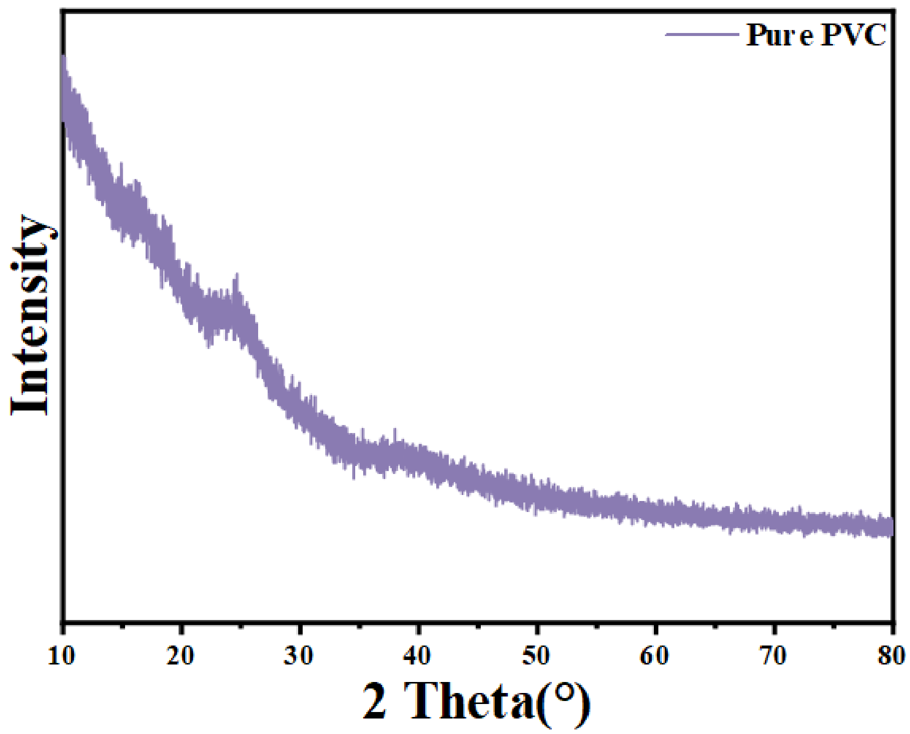

Fig.S5. XRD patterns of PVC

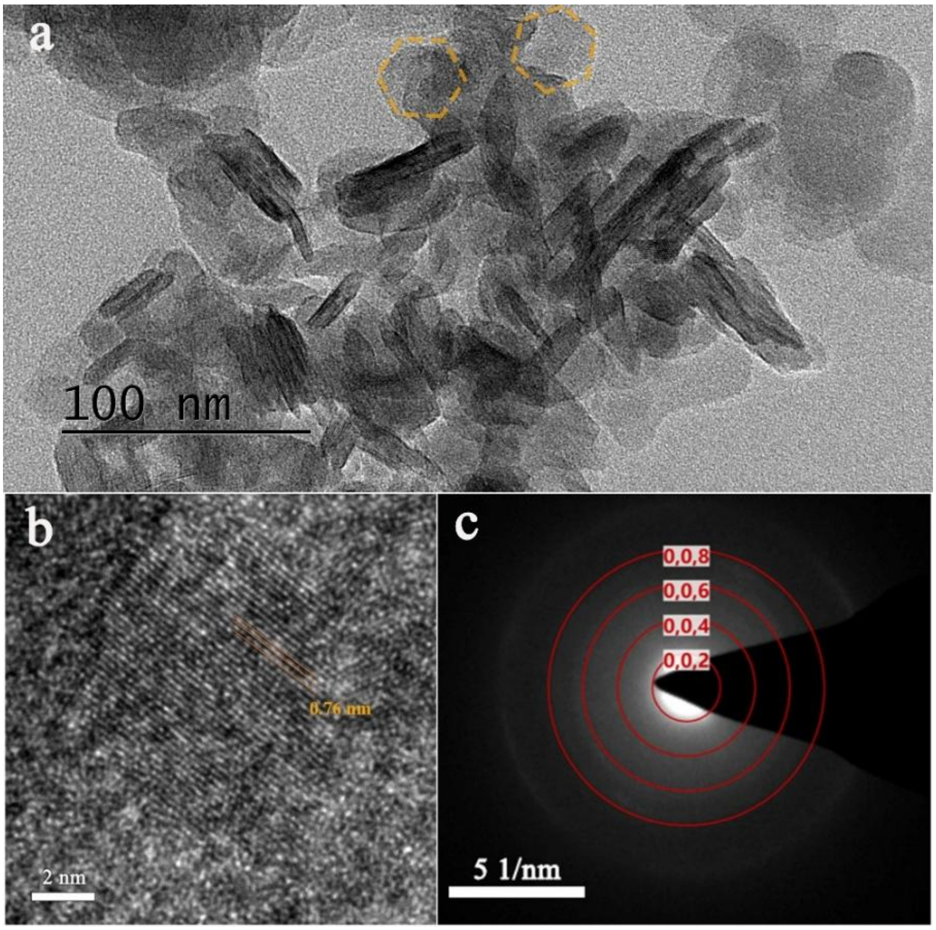

Figure S6. TEM images (a), HRTEM image (b), SAED pattern (c) of Li/Al-LDH.

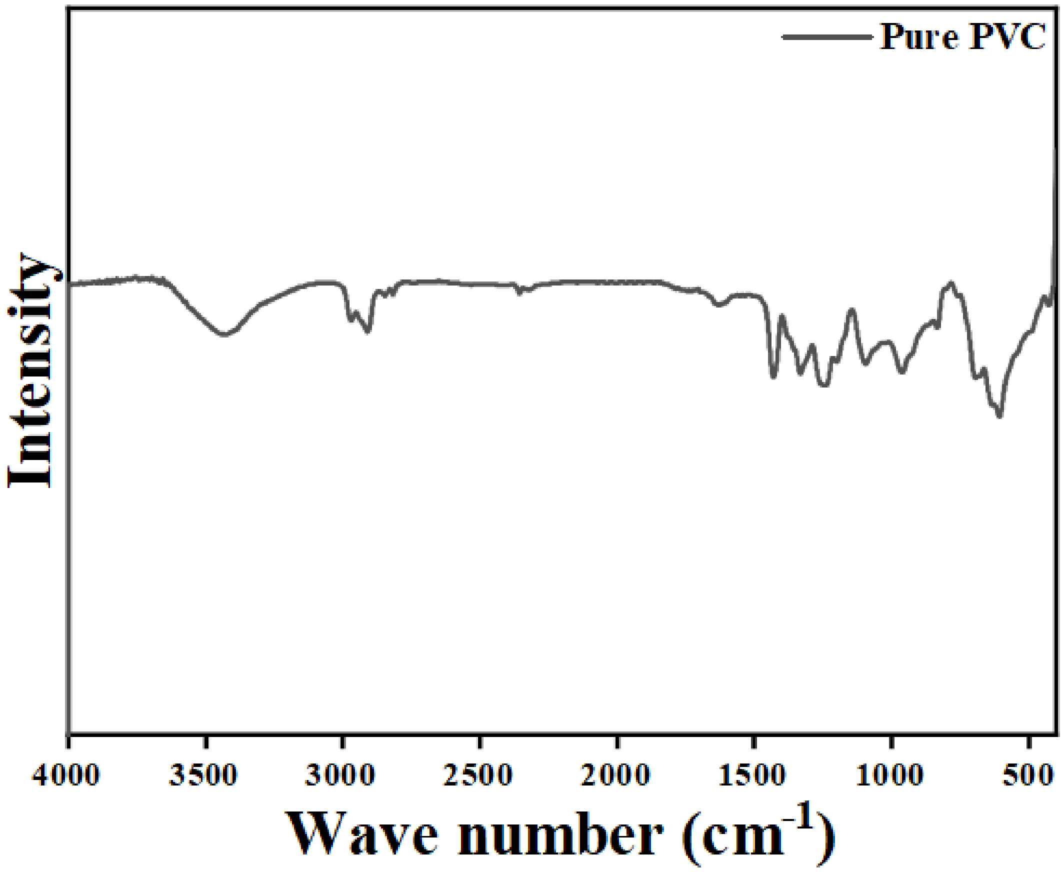

Fig.S7. FT-IR spectrum of PVC

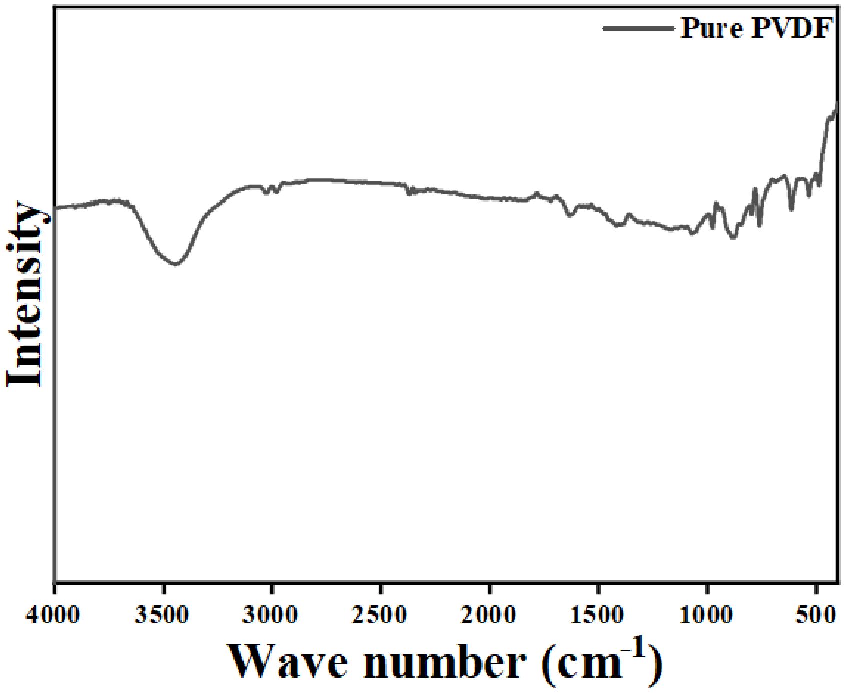

Fig.S8. FT-IR spectrum of PVDF

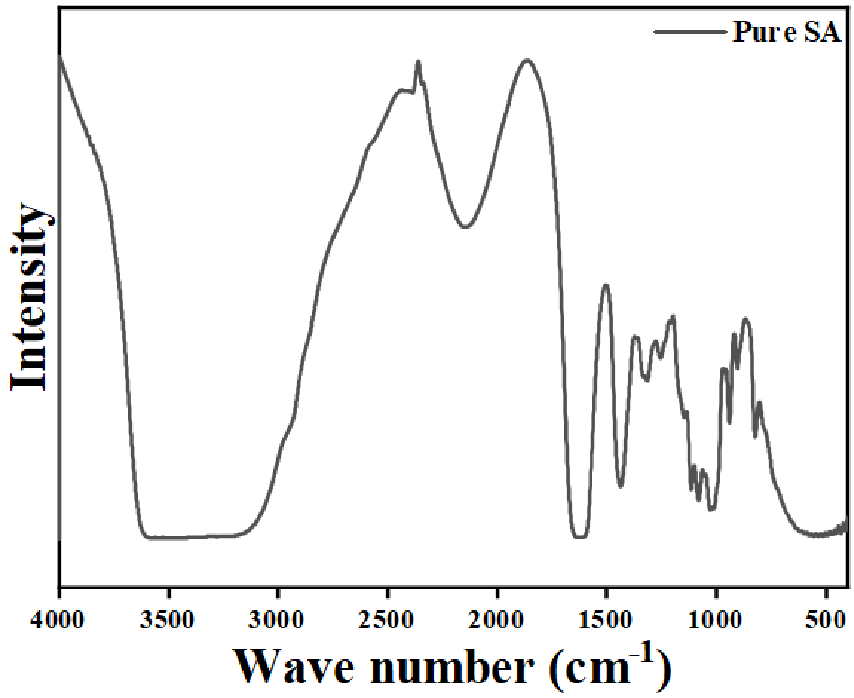

Fig.S9. FT-IR spectrum of SA

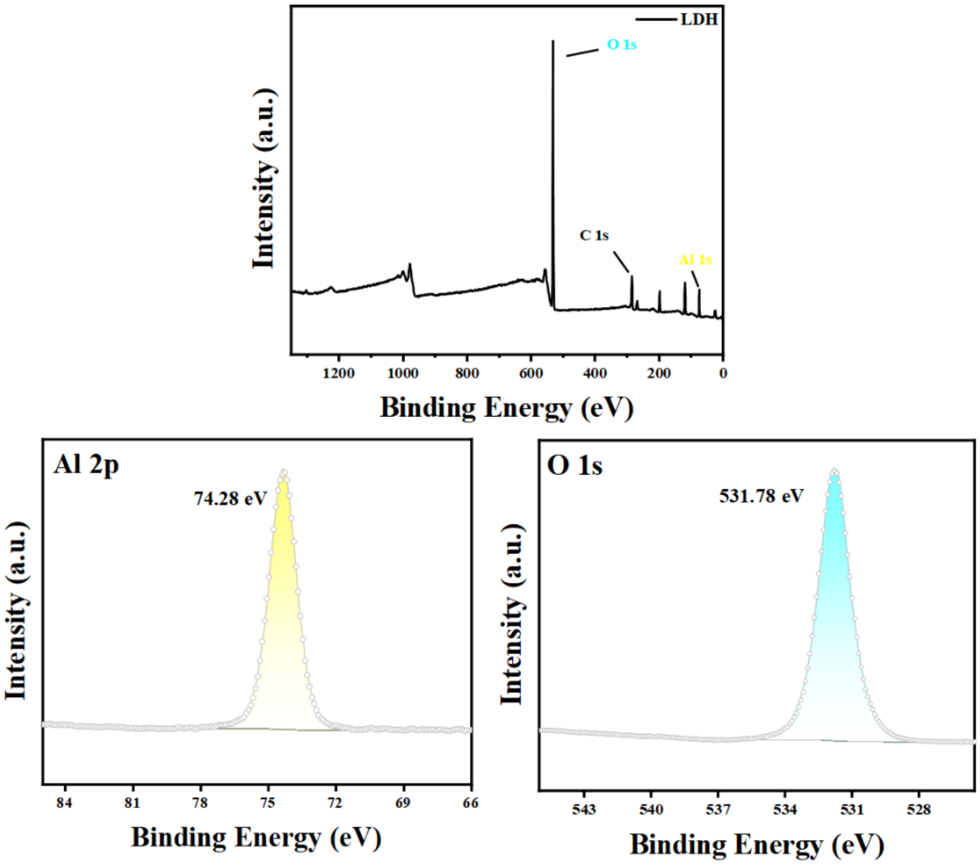

Figure S10 XPS survey spectrum of Li/Al-LDH.

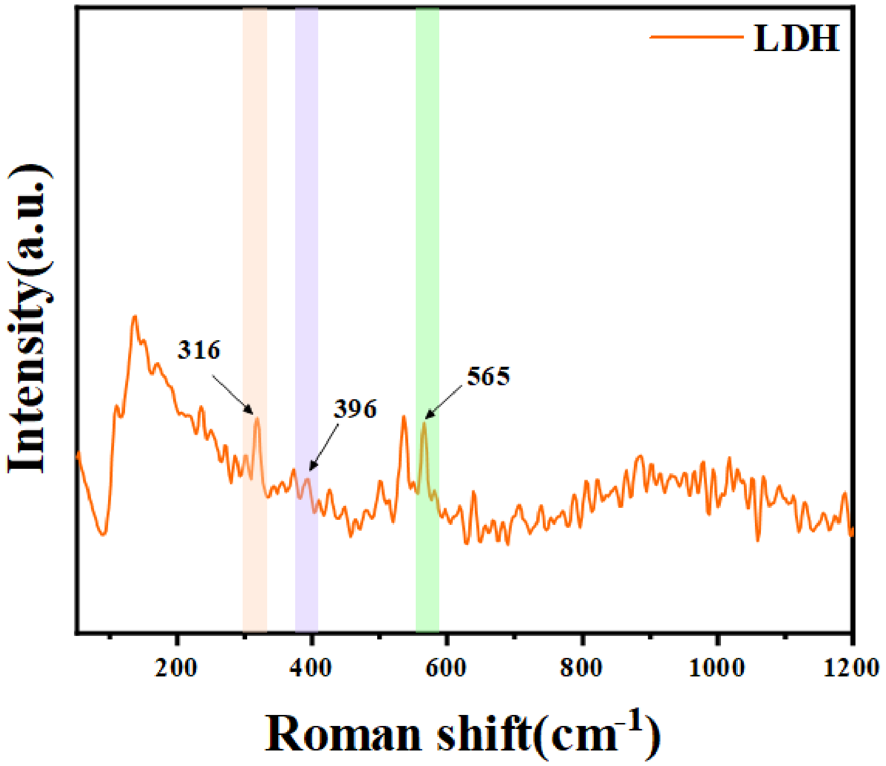

Figure S11. Raman spectra of Li/Al-LDH.

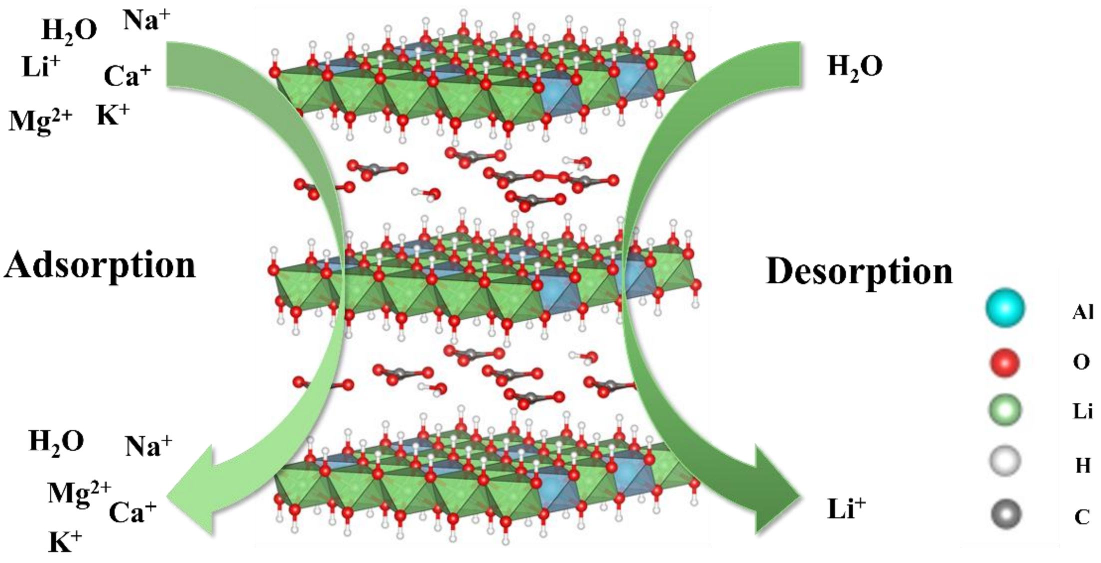

Figure S12. The structure and lithium extraction of Li/Al-LDH.

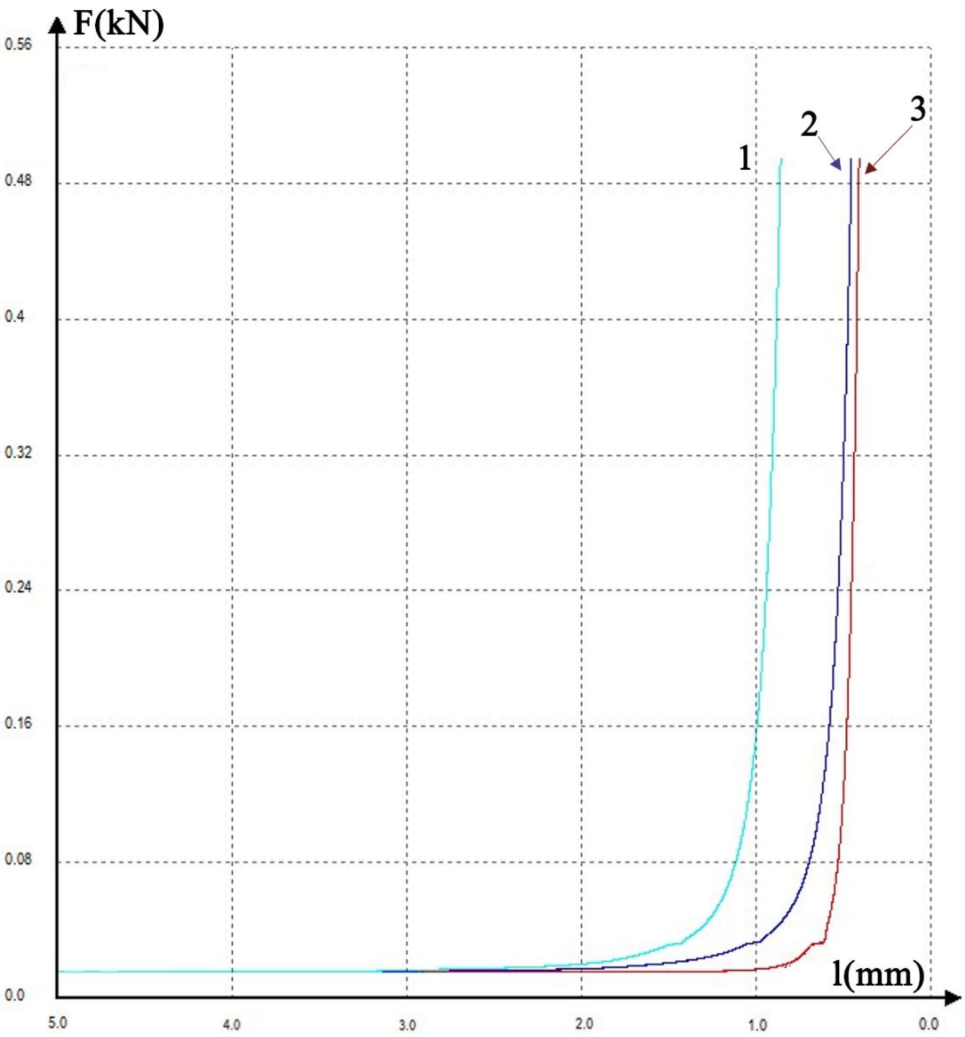

Figure S13. Compression performance test chart of PVC-LDH(1), PVDF-LDH(2), and SA-LDH(3).

Table S1. Kinetic parameters

Qe（mg/g）	伪一级动力学模型			伪二级动力学模型		
	k_1	Qe_1(mg/g)	R_2	k_2	Qe_2(mg/g)	R_2
3.76918	0.3990	4.3921	0.7920	0.0158	3.8423	0.9928

Table S2. Particle size distribution of aluminum-based lithium adsorbent

Dx（10）	Dx（50）	Dx（90）
17.0 μm	57.9 μm	155 μm
